# Screen time and pulmonary function in hospitalized children with cystic fibrosis

**DOI:** 10.36416/1806-3756/e20250284

**Published:** 2026-03-05

**Authors:** Vanessa dos Santos Rodrigues, Caroline Schmidt, Gleice Kelly Medeiros, Guilherme Hoff Affeldt, Bruna Ziegler

**Affiliations:** 1. Programa de Saúde da Criança, Hospital de Clínicas de Porto Alegre - HCPA - Universidade Federal do Rio Grande do Sul - UFRGS - Porto Alegre (RS) Brasil.; 2. Programa de Pós-Graduação em Ciências Pneumológicas, Universidade Federal do Rio Grande do Sul - UFRGS - Porto Alegre (RS) Brasil.; 3. Serviço de Fisioterapia, Hospital de Clínicas de Porto Alegre - HCPA - Universidade Federal do Rio Grande do Sul - UFRGS - Porto Alegre (RS) Brasil.

**Keywords:** Screen time, Spirometry, Cystic fibrosis, Child, hospitalized

## Abstract

**Objective:**

To evaluate the relationship between screen time (ST) and lung function in hospitalized children and adolescents with cystic fibrosis (CF).

**Methods:**

This was a cross-sectional study of 45 children with CF in the 0- to 17-year age bracket admitted to a public hospital in southern Brazil. ST during hospitalization was measured by means of a recall diary. Questionnaires were used in order to collect data on screen use and personal data. Data on lung function, nutrition, bacteriology, and the Shwachman-Kulczycki clinical score were obtained from patient medical records.

**Results:**

Of the study participants, 51.1% were male, with a median age of 9 years, and 80% were White. The mean FEV_1_ (Z-score) was −2.9 ± 1.9, and FEV_1_ (in % of predicted) was 63.7 ± 22.3. The median ST was 315 min, and 95.5% of the study participants exceeded the recommended ST. The most prevalent reasons for using electronic devices during hospitalization were boredom and lack of other activities. In a multivariate analysis, ST (the dependent variable) was significantly associated with age (b = 26.591; 95% CI, 14.695-38.487), time spent watching television at home (b = 0.686; 95% CI, 0.304-1.069), and FEV_1_ Z-score (b = −60.631; 95% CI, −115.399 to −5.864).

**Conclusions:**

Excess ST appears to be associated with worse lung function in hospitalized children with CF, as do older age and longer periods of time spent watching television at home.

## INTRODUCTION

The advancement of the digital age has led to an increasing number of children and adolescents having access to electronic devices.^1^ Time spent using screens (such as smartphones, tablets, computers, and television sets) is referred to as “screen time” (ST) and is considered a subtype of sedentary behavior.^2^ In fact, ST is one of the most common forms of sedentary behavior among young people worldwide, highlighting the growing access to technology that has been evident since the beginning of the century.^3^

Excessive screen and media use has been studied in children and adolescents. Research suggests that uncontrolled media consumption is associated with various health issues, including mental health disorders such as anxiety and depression; sleep and self-esteem disturbances; eating disorders such as obesity and bulimia; physical inactivity; and increased exposure to violence and substance use.^4^

Several national and international organizations have addressed this issue to raise awareness and prevent health problems in children and adolescents, providing guidelines for safer media use.^4^ ^-^ ^8^ The WHO advises that children < 1 year of age should not be exposed to screens; those in the 1- to 4-year age bracket should be limited to a maximum of 1 h per day; and those ≥ 5 years of age, including adolescents, should have a ST limit of 2 h per day.^6^ ^,^ ^7^

Cystic fibrosis (CF) is a chronic genetic disease that affects multiple systems, including the respiratory system. It leads to increased mucus viscosity, making it harder to clear, which in turn promotes infection by microorganisms and airway obstruction, ultimately contributing to lung disease.^9^ An active lifestyle, including regular physical exercise, not only helps clear mucus from the airways but also improves exercise capacity and lung function, both of which are protective factors for individuals with CF.^10^ ^,^ ^11^

Therefore, it is important for pediatric CF patients to be encouraged to maintain an active, rather than sedentary, routine, even in the hospital setting, which can also be an opportunity to promote healthier habits and provide guidance on a more active lifestyle. Previous studies^12^ ^-^ ^14^ involving hospitalized pediatric populations have shown that ST in the hospital setting is excessive and superior to ST at home. To date, there have been no studies evaluating ST in CF patients in the hospital setting or at home, representing an important knowledge gap that requires further study. The objective of the present study was to evaluate the relationship between ST and lung function in hospitalized children and adolescents with CF. 

## METHODS

This was a cross-sectional epidemiological study.^15^ The study was approved by the Research Ethics Committee of the *Hospital de Clínicas de Porto Alegre* (HCPA), located in the city of Porto Alegre, Brazil (Protocol no. 2019-0670). The study was conducted in accordance with Brazilian National Health Council Resolution no. 466/2012. Participants were conveniently selected from the HCPA Pediatric Inpatient Unit. 

**Figure 1:**
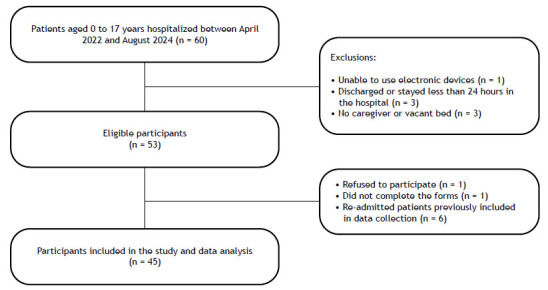
Participant selection flow chart.

Inclusion criteria were as follows: CF patients in the 0- to 17-year age bracket hospitalized in the HCPA Pediatric Inpatient Unit during data collection (from April of 2022 to August of 2024). Patients without a companion or those with conditions that prevented the use of electronic devices were excluded from the study. Figure 1 illustrates the participant selection process. 

Parents were asked to sign an informed consent form in accordance with Brazilian National Health Council Resolution no. 466/2012, which regulates research involving human subjects.^16^

Personal and hospitalization data were collected via a questionnaire designed by the authors. The questionnaire gathered data on ST in the hospital and home settings, as well as on the context and reasons for using electronic devices, including clinical and emotional factors. 

ST exposure was assessed by means of a recall diary, in which the accompanying person recorded the start and end times of each period during which the patient was exposed to electronic devices (including smartphones, tablets, television sets, video games, and DVD players) over a 24-h period during hospitalization, along with the name of the program being watched. The recall diary was applied in the first 48 h of hospitalization. 

Regarding device use, participants were categorized as having “met” or “not met” the recommendations for their age group. The cutoff points were as follows: no ST for children < 1 year of age; up to 1 h per day for those in the 0- to 4-year age bracket; and a maximum of 2 h per day for those ≥ 5 years of age, including adolescents.^6^ ^,^ ^7^

Personal and hospitalization data were collected via questionnaires and by reviewing electronic medical records, with data on reasons for hospitalization, diagnoses, comorbidities, and interventions performed during the hospital stay being collected from the latter. 

Spirometry was performed at the Pulmonary Physiology Unit of the HCPA Department of Pulmonology. The test was performed with a MasterScreen v4.31a spirometer (Jaeger Medical, Höchberg, Germany), with patients in a sitting position, being performed in accordance with the acceptability criteria outlined in the 2002 Brazilian Thoracic Association Guidelines for Pulmonary Function Tests.^17^ FEV_1_, FVC, and the FEV_1_/FVC ratio were measured. The values were expressed as Z-scores and as a percentage of the predicted values based on sex, age, and height.^17^ The most recent spirometry test performed during a period of disease stability before hospitalization was recorded. 

Sputum bacteriology was performed at the HCPA Department of Microbiology. Bacteria identified by tests conducted in the last 12 months were recorded. 

BMI was calculated by dividing the total body mass in kilograms (weight) by the square of height in meters. BMI was expressed in kg/m^2^ and as a Z-score based on the WHO reference curve.^18^

The socioeconomic status of participants was determined by using the Brazilian Market Research Association economic criteria. The categories (classes A to E) range from the highest to the lowest average monthly household income (i.e., from 21,826.74 Brazilian reals for class A to 900.60 Brazilian reals for class E).^19^ These classes were further stratified and analyzed as follows: high socioeconomic status (classes A and B) and low socioeconomic status (classes C, D, and E). 

The Functional Status Scale is an assessment tool designed to evaluate functioning in hospitalized pediatric patients. It has been translated into Portuguese and validated for use in Brazil.^20^ It consists of six domains: mental status, sensory, communication, motor, feeding, and respiratory. Each domain is scored on a scale from 1 (normal) to 5 (severe dysfunction). The total score ranges from 6 to 30 points, with lower scores indicating better functioning.^20^ ^,^ ^21^

The short version of the International Physical Activity Questionnaire includes open-ended questions regarding activities performed in the week prior to the survey.^22^ ^,^ ^23^ The International Physical Activity Questionnaire classifies individuals as sedentary, insufficiently active (A and B), active, or very active.^22^ In the present study, the questionnaire was administered only to individuals > 6 years of age. For the analysis, two groups were created: “sedentary” (individuals pre-classified as sedentary or insufficiently active A or B) and “active” (individuals classified as active or very active). 

The Shwachman-Kulczycki score is a clinical assessment system that evaluates four different characteristics (general activities, physical exam, nutrition, and chest radiological findings); each characteristic is scored by the evaluator on a scale from 5 to 25 points (with higher scores indicating better performance). A final score of 100 points shows that a patient is in excellent clinical condition.^24^

Data were entered into an Excel spreadsheet, and statistical analysis was performed with the IBM SPSS Statistics software package, version 18.0 (IBM Corporation, Armonk, NY, USA). Qualitative variables were described as frequencies and proportions. Symmetric quantitative variables were described as means and standard deviations, whereas asymmetric variables were described as medians and interquartile ranges. The Shapiro-Wilk test was used in order to assess the normality of the quantitative variables. Simple linear regression analyses were performed to determine associations between variables. Two regression models were designed, with ST being used as the dependent variable. Model 1 included all participants, whereas model 2 included only children > 6 years of age, given that only children in that age group undergo spirometry for respiratory function testing. Multicollinearity was assessed. The regression coefficient (b) was calculated with a 95% confidence interval, and a 5% significance level was set (p < 0.05). All statistical analyses were performed with the IBM SPSS Statistics software package, version 18.0 (IBM Corporation, Armonk, NY, USA). 

## RESULTS

Our study included 45 hospitalized children and adolescents with CF. Of those, 36 (80%) were White and 23 (51%) were male. The median age was 9 years. In our sample, 12 (26.7%) were classified as active. Table 1 presents the general characteristics of the study participants. 

**Table 1:** General characteristics of hospitalized children with cystic fibrosis.^a^

Variable	n = 45
Male	23 (51.1%)
White	36 (80%)
Age, years	9 [10]
BMI (kg/m^2^)	16.4 ± 2.1
BMI (Z-score)	−0.49 [1.6]
Length of hospital stay, days	15 [5]
Physical activity level	
Active	12 (26.7%)
Sedentary	15 (33.3%)
Not applicable	18 (40%)
Socioeconomic status	
High	10 (22.2%)
Low	35 (77.8%)
FSS score	6 [1]
Shwachman-Kulczycki score	75 [30]
FEV_1_ (Z-score)	−2.9 ± 1.9
FEV_1_ (% predicted)	63.7 ± 22.3
FVC (Z-score)	−2.2 ± 1.9
FVC (% predicted)	74.7 ± 21.2
FEV_1_/FVC (Z-score)	−1.7 ± 1.3
FEV_1_/FVC (% predicted)	84.1 ± 14
MSSA	39 (86.7%)
MRSA	6 (13.3%)
*Pseudomonas aeruginosa*	29 (64.5%)
*Burkholderia cepacia*	7 (15.6%)

FSS: Functional Status Scale; MSSA: methicillin-sensitive *Staphylococcus aureus*; and MRSA: methicillin-resistant *Staphylococcus aureus*. ^a^Values expressed as n (%), mean ± SD, or median [IQR].

Regarding access to electronic devices, all beds in the HCPA Pediatric Inpatient Unit are equipped with a television set and Wi-Fi access. Most (n = 29; 64.5%) of the caregivers reported that the child or adolescent spent more time using electronic devices during hospitalization than the caregiver would have liked them to. The most common reasons for ST during hospitalization were boredom, in 29 (64.5%); lack of other activities, in 25 (55.6%); and excessive crying or complaints, in 19 (42.2%). Most (n = 40; 88.9%) of the caregivers also stated that they believed that it was important to limit ST. However, 33 (73.3%) reported that electronic devices helped alleviate patient discomfort, and 14 (31.1%) said that the devices helped at mealtimes. The median ST over a period of 24 h, as measured by using a recall diary, was 315 min, with only 2 (4.4%) of the study participants adhering to the WHO recommendations. Information on ST exposure is shown in Table 2. 

**Table 2:** Factors relating to screen exposure in children with cystic fibrosis in the hospital setting.^a^

Variable	n = 45
Reasons for electronic device use	
Busy caregiver	8 (17.8%)
Pain	6 (13.3%)
Severity of illness	6 (13.3%)
Boredom	29 (64.5%)
Anxiety	15 (33.3%)
Lack of other activities	25 (55.6%)
Presence of hospital equipment	10 (22.2%)
Excessive crying or complaints	19 (42.2%)
Falling asleep	7 (15.6%)
Playing	4 (8.9%)
Learning	14 (31.1%)
Helping with discomfort in the hospital	33 (73.3%)
Assisting with meals	14 (31.1%)
Caregiver perception of screen time	
Above desired	29 (64.5%)
Adequate	13 (28.9%)
Below desired	3 (6.7%)
24-h screen time diary, min	315 [348]
WHO classification	
As recommended	2 (4.4%)
Above recommended	43 (95.6%)

a Values expressed as n (%) or median [IQR].

In the present study, caregivers were asked about child ST in the home. The most widely used device was a smartphone, in 37 (82.2%), followed by television, in 36 (80%). Noneducational content was viewed by 17 (37.8%) of the study participants. Of the caregivers, 29 (64.5%) reported that their child spent more time using electronic devices in the home than the caregiver would have liked them to.

Table 3 presents our linear regression model with ST as the dependent variable. The univariate analysis showed a significant association between ST and age, as well as between ST and time spent watching television at home during the week (p < 0.05). Those associations remained significant in the multivariate analysis. 

**Table 3:** Univariate and multivariate linear regression models including screen time as the dependent variable (n = 45).

	Model 1					
	Univariate			Multivariate		
Variable		CI	p	b	CI	p
Sex	88.563	−74.291 to 251.417	0.279			
Age, years	31.599	18.517-44.681	< 0.001	26.591	14.695-38.487	< 0.001
Socioeconomic status	40.100	−158.048 to 238.248	0.685			
Caregiver’s level of education	14.035	−164.200 to 192.269	0.875			
Number of children	−36.309	−114.631 to 42.014	0.355			
Time spent watching TV at home during the week	0.887	0.440-1.335	< 0.001	0.686	0.304-1.069	0.001
FSS score	67.293	−12.798 to 147.384	0.097			

FSS: Functional Status Scale; and b: regression coefficient.

Table 4 presents our linear regression model for children > 6 years of age. In the univariate analysis, age, clinical score, and FEV_1_ were significantly associated with the dependent variable (i.e., ST; p < 0.05). In the multivariate analysis, age and FEV_1_ remained significant (p < 0.05). 

**Table 4:** Univariate and multivariate linear regression models including screen time as the dependent variable (n = 30).

	Model 2					
	Univariate			Multivariate		
Variable	b	CI	p	b	CI	p
Age, years	42.596	17.066-68.125	0.002	38.038	17.035-59.040	0.001
FEV_1_ Z-score	−83.858	−131.410 to −36.307	0.001	-60.631	−115.399 to −5.864	0.031
Shwachman-Kulczycki score	−7.590	−12.977 to −2.204	0.007	−2.394	−8.224 to 3.436	0.406
MSSA	−169.596	−473.783 to 134.591	0.263			
MRSA	117.673	−190.166 to 425.512	0.440			
*Pseudomonas aeruginosa*	47.304	−202.134 to 296.743	0.701			
*Burkholderia cepacia*	−51.404	−300.721 to 197.913	0.676			

MSSA: methicillin-sensitive *Staphylococcus aureus*; MRSA: methicillin-resistant *Staphylococcus aureus*; and b: regression coefficient.

## DISCUSSION

In the present study, we evaluated the relationship between ST and lung function in hospitalized children and adolescents with CF. Our results show that ST was significantly associated with FEV_1_, age, and time spent watching television at home. More than 95% of the children did not meet the WHO ST recommendations, with a median of 315 min spent in front of screens over a 24-h period. The most common reasons for using electronic devices during hospitalization were boredom, lack of alternative activities, and excessive crying or complaints. 

We found that better lung function was associated with reduced ST, as an increase of 1 standard deviation in FEV_1_ was linked to a 60-min decrease in ST. One possible explanation is that children with more severe lung disease tend to be more confined to bed, leading to longer ST, whereas those who are less compromised have more energy to remain active. To date, there have been no studies investigating the association between ST and lung function in individuals with CF. 

Although ST is commonly used as a marker of physical inactivity in pediatric studies and is consistently associated with lower physical activity and poorer fitness outcomes,^25^ ^-^ ^28^ it does not fully capture total sedentary behavior, which includes non-screen-based activities.^29^ Our findings regarding ST should be interpreted within this broader context. Quality improvement initiatives in pediatric wards, such as staff and caregiver education, combined with screen-free alternative activities, have led to substantial reductions (of approximately 37%) in ST among hospitalized children, without increasing staff burden.^30^ Protocols involving structured play interventions have also demonstrated practical value in replacing ST with routine, play-based activities.^31^ Behavioral interventions based on goal setting, feedback, and planning have shown moderate effectiveness and, in some cases, reductions in ST ranging from 30% to 90%.^32^ It is worth noting that digital media can be beneficial when used as a therapeutic tool-such as for distraction during painful procedures or for preparation using virtual reality-provided that it is balanced with appropriate non-digital alternatives.^12^

We found that older children tended to have higher ST. This finding has been reported by other authors, such as those of a study examining ST in hospitalized children and adolescents in Brazil.^33^ The study showed that each additional year of age increased the likelihood of watching television by 93.85%.^33^ In a study examining screen media use in hospitalized children and adolescents in the U.S., it was found that the older the patient, the greater the probability of screen exposure.^12^ This can be explained by the greater independence that children gain as they grow older, having more control over their daily activities, including ST. Additionally, as children age, they are more likely to own personal digital devices rather than relying on those provided by their caregivers, a trend observed in the 2023 Brazilian Institute of Geography and Statistics survey.^34^

In our study, longer periods of time spent watching television at home were associated with increased ST during hospitalization. This suggests that screen habits at home persist in the hospital setting. Similar findings were reported by Martins & Bacellar,^33^ who found that children and adolescents who regularly watched television at home were more likely to do so in the hospital setting. The same pattern was observed for other electronic devices. In our sample, 95.6% of the hospitalized CF patients exceeded the maximum ST recommended by the WHO,^6^ ^,^ ^7^ with a median daily ST of 5 h 25 min. Excessive ST is known to be associated with various health issues in children and adolescents, including anxiety, depression, sleep disorders, and obesity, as well as physical inactivity and sedentary behavior.^4^ ^,^ ^35^ Supporting our findings, another study showed that most hospitalized children and adolescents in the 0- to 18-year age bracket exceeded the recommended ST.^33^ The study included 40 participants and analyzed ST and electronic device use at home and in the hospital setting from the perspective of caregivers via questionnaires.^33^ The authors found that weekday and weekend times spent watching television at home were similar to the time spent watching television in the hospital setting and that ST across other electronic devices exceeded the recommended limits in both settings.^33^

Excessive screen exposure among children and adolescents has emerged as a significant concern in pediatric health care because of its associations with cognitive, behavioral, and physical health risks. The Brazilian Society of Pediatrics has recently issued evidence-based guidelines^36^ that align with recommendations from international bodies such as the WHO and the American Academy of Pediatrics,^6^ ^,^ ^37^ while emphasizing contextual factors relevant to the Brazilian population. The Brazilian Society of Pediatrics advises complete avoidance of screen exposure for children < 2 years of age; a maximum of 1 h per day for those in the 2- to 5-year age bracket; and limited screen times of 1-2 h and 2-3 h per day for children in the 6- to 10-year age bracket and adolescents in the 11- to 18-year age bracket, respectively, all under active parental supervision.^36^ The guidelines also discourage screen use during meals and before bedtime.^36^ In pediatric hospital settings, such recommendations have direct implications for clinical policy, suggesting the need for structured ST protocols; supervised and age-appropriate media use; and promotion of alternative therapeutic activities such as storytelling, art, and physical play. Integrating these principles into inpatient care can help mitigate screen-related health risks while supporting holistic child development and family education during hospitalization. 

Regarding the reasons for excessive ST during hospitalization, more than half of the caregivers in the present study cited boredom as a key factor. Other frequently mentioned reasons included lack of alternative activities, excessive crying or complaints, and anxiety. In a study including 96 hospitalized children in the 4-month to 20-year age bracket, screens were reported to serve as a means of escape from boredom, distress, and loneliness during hospitalization.^12^ Similarly, in a study conducted in a tertiary care hospital and involving 254 hospitalized patients in the 0- to 18-year age bracket, it was found that ST was used in order to help children relax, and the absence of alternative activities was associated with increased ST.^13^ Hospitals provide limited resources for promoting activities among children. In a study conducted in northern India,^38^ the authors found a lack of internal policies and designated schedules for encouraging play in a tertiary hospital. Additionally, overburdened health care professionals focused on clinical care and provided little stimulation for pediatric patients to engage in recreational activities.^38^

One of the limitations of our study is its cross-sectional design, which does not allow for inferences over time. Another limitation is the self-report nature of the recall diary used to record ST; because this is a subjective measure, it may be underestimated by the observer. In addition, our sample was small because of low hospitalization rates in the pediatric population. Furthermore, ours was a convenience sample, which does not allow generalization of the results for this population. On the other hand, one strength of the present study is its contribution to a better understanding of screen exposure, a highly relevant issue today. We believe that our study can foster reflection and support the development of future measures in hospital settings to guide patients and their families regarding ST. Additionally, our analysis of the association between ST and other important factors affecting the lives of CF patients adds significant value to research. 

The present study showed that hospitalized children with CF exceed acceptable limits of use of electronic devices. Excessive ST in children with CF is associated with older age, poorer lung function, and longer periods of time spent watching television at home prior to hospitalization. Encouraging the development of policies to regulate electronic device use in hospitals may help modify these habits. Moreover, implementation of more engaging and recreational activities provides a feasible, low-cost alternative aimed at keeping children more active and reducing their ST. 
